# Lactating Mothers’ Perception Toward Diarrheal Disease in Bench-Maji Zone, Southwest Ethiopia: Mixed study design

**DOI:** 10.11604/pamj.2018.31.176.11204

**Published:** 2018-11-13

**Authors:** Wadu Wolancho Debancho, Abraham Tamirat Gizaw, Fira Abamecha Ababulgu

**Affiliations:** 1 Department of Nursing and Midwifery, Faculty of Health Science, Institute of Health, Jimma University, Ethiopia; 2 Department of Health, Behavior and Society, Faculty of Public Health, Institute of Health, Jimma University, Ethiopia

**Keywords:** Perception, Knowledge, lactating mothers, diarrheal disease, Susceptibility Maji

## Abstract

**Introduction:**

Acute diarrheal diseases are the leading cause of preventable death, especially among children under-five in developing countries. Worldwide and nationwide diarrheal disease is the second leading cause of death in under-five year children. Therefore, the aim of this study is to assess perception of lactating mothers' toward diarrheal disease in Mizan-Aman District, South-West Ethiopia.

**Methods:**

Community based cross-sectional quantitative study supplemented by qualitative study was employed. A total of 383 selected households with the lactating mothers were involved in the study. Data was collected through face-to-face interview technique by trained data collectors. Data was entered and analyzed using SPSS version 16. Multiple logistic regressions analysis was used to identify the independent predictors. Odds ratio, with 95% confidence level and P < 0.05 were used to determine statistically significant association.

**Results:**

The majority of the respondents had primary education (44.4%) and from rural area (52.2%). Multiple logistic regression analysis showed that the residence area [AOR = 4.79, CI (1.33,7.78), P<0.003], educational status [AOR = 0.72, CI (0.55,1.29), P<0.045], Wealth index [AOR = 8.9, CI (0.99,17.45), P<0.001], knowledge [AOR = 2.34, CI (1.2-4.3) P<0.023]. Perceived susceptibility [AOR = 0.44, CI (0.33,11.33), P<0.001] and perceived severity of their child towards diarrheal disease [AOR= 0.24, CI (1.23,7.99), P<0.033] had significant associations with the perception of the diarrheal diseases among lactating mothers.

**Conclusion:**

Lactating mothers' perceptions toward their children of getting diarrheal disease and danger of the disease with primary education and above were better protected than mothers with no education. Thus, implementing effective information educational communication (IEC) programs that emphasize on the benefit of complying with nationally recommended practice to prevent diarrheal disease is important to reduce the risk.

## Introduction

Globally acute diarrheal disease remains a major public health problem. In developing countries, an estimated 12 or more diarrheal episodes per child per year occur within the first 5 years of life. Every year, approximately 4.6 million pediatric deaths, about 25 to 30% of all deaths among children less than age 5 years, can be attributed to acute diarrhea. According to WHO and UNICEF report almost 2.5 billion episodes of diarrhea occur annually in children under five years of age in developing countries with more than 80% of the episodes occurring in Africa and South Asia (46% and 38% respectively) [1]. The study showed in sub-Saharan African countries children under-five years of age experience about five episodes of diarrhea each year. The analysis also showed that prevalence of childhood diarrhea ranged from 10.5 to 19%. The risk factors contributing to diarrheal disease were age of the child, quality and quantity of water, availability of toilet facilities, housing conditions, maternal level of education, household economic status, place of residence, feeding practices and general sanitary condition around the house [2, 3]. Study conducted in Eritrea shows that availability toilet facility in the household was associated with 27% reduction of diarrhea in under-five children. The prevalence of diarrhea in under-five children in Botswana is 10% and 40% each in Senegal and Liberia. Each child in sub- Saharan Africa has 5 episodes of diarrhea per year and 8 million die each year due to diarrhea and dehydration [4, 5].

A study done in indigenous and resettlement community in Assosa district showed that 94.7% mothers from indigenous community and 88.1% of mothers from settlement community reported diarrhoea to be a "serious health problem." The difference between responses from participants of the two areas was not, however, statistically significant. Other finding from Finote Gojam, Ethiopia showed that the maternal educational status showed significant association with the childhood diarrheal management diarrheal management [6, 7]. According to the 2012 Ethiopian Demographic and Health Survey (EDHS 2012), Diarrhoea was most common among children age 6-23 months (23-25 percent). Diarrhoea prevalence is highest among children residing in households that drink from unprotected wells (18 percent), those residing in rural areas (14 percent) and children residing in Benshangul-Gumuz and Gambella (both 23 percent) [8]. Recent studies at the national level tried to assess knowledge; attitude and practice about diarrheal disease, treatment seeking and preventive measures but studies on household perceptions specially on lactating mothers are limited. This will call further research efforts since diarrheal disease related perception varies not only among different cultures but also among individuals depending on their socioeconomic background.

## Methods

**Study setting and design:** Community based cross-sectional study conducted during March 1-30, 2013 in Mizan-Aman district. Maji district is one of the nine districts of Bench Maji Zone, Southwest of Ethiopia, located 550km away from the capital Addis Ababa.

**Study participants:** All randomly selected households with the lactating mothers in Mizan-Aman District were included. Household lactating mothers were used as study participants. Households resided in the study area for more than six month and respondents who could communicate were included.

**Sample size and sampling technique:** The sample size was determined using single population proportion formula, taking the following parameters: single population proportion (P = 13%), P which is prevalence of diarrheal disease for under-five children at national level reported by DHS in 2011 [9], 95% confidence interval and 5% marginal error of the estimate was considered. This yields a sample of 174. To ensure the design effect of multi-stage sampling technique 174×2 = 348 maintaining 10% non-response rate, the final sample size became 383. For qualitative study, 19 key respondents were interviewed until saturation and redundancy of information occurred. Within district; there are small administrative entities called kebeles. There were a total of twenty-three kebeles in Mizan-Aman district. Ten kebeles were selected randomly using list of kebeles as sampling frame. The numbers of study households from each selected kebele were determined by proportional to the number of households in the kebele. Finally, households were selected by simple random sampling technique using lists the household as sampling frame.

**Data collection:** Structured questionnaires were adapted from previous similar studies as per the local context [9-11]. The instrument was prepared in English, then translated to official language (Amharic) and finally back translated to English by different individuals to keep consistency. The instrument covered information on socio-demographic status, knowledge on diarrheal disease in under five years, perception of lactating mothers on prevention of diarrheal disease. Data was collected through face-to-face interview of lactating mothers in household using Amharic version instrument. Three trained diploma nurses were collected. Two supervisors with master's holders in health sciences and principal investigator supervised the whole process of data collection. For qualitative data, in depth interview guide was prepared for judgmentally selected household who have lactating their child to collect information by exploring on the perception toward diarrheal disease, Diarrhea related specific beliefs and perception. Qualitative respondents were not included in quantitative samples. Individuals from five kebeles were involved on the in-depth interview. On average; single in-depth interview was taking 49 minutes. The principal investigator conducted in-depth interview using tape recorder.

**Statistical data analysis:** After data collection, data entry was done using EPI INFO version 3.5 statistical packages. Frequency output used to check missing values and outliers. Descriptive statistics and summary measures were employed to the data and cleaning was done using original code number. Using odds ratio (OR) with 95% limit of confidence interval, the association of dependent and independent variables were assessed and their degree of associations will be computed. Potential confounding variables were controlled by using Multiple Logistic regression using SPSS version 16 statistical packages. Qualitative data was transcribed in the original language of interview first word-by-word from the audio tapes and field notes. And then it was translated to English for analysis. Primary theme was produced through manual coding using pen of different colors. Then it was pooled into broader concepts to form main themes. Besides, quotes of participants that exemplify key concepts were used directly during analysis. Finally, the result was triangulated with that of the quantitative one.

**Data quality management:** Study questionnaires were adapted from previous similar studies as per the local context. The instrument was prepared in English first, then translated to Amharic and finally back translated to English by different individuals to keep consistency. Training was given for qualified data collectors and supervisors. The entire process of data collection was assisted and guided by supportive and close supervision. The collected data were checked frequently at the field by the supervisors for its completeness and consistency. Pretesting on 5% of respondents outside the study area but of the similar background was done to for cultural sensitivity and clarity of the questionnaire.

## Results

**Socio-demographic characteristics:** A total of 383 lactating mothers where sampled using Fisher's formula. Two hundred sixty-seven (69.7%) were Orthodox Christians while 12(3.1%) were Muslims. Almost all respondents were married (96.9%) and housewife (37.6%), most had primary education (44.4%) and were from rural area (52.2%). Mean month of the respondent after giving birth was 19.6 ± 12.5 months (Table 1).

**Table 1:** Socio-demographic characteristics of lactating mothers in Mizan-Aman district, South-West Ethiopia, March, 2013 (n=383)

Variable	category	Frequency	Percent
Age in years	15-19	25	6.5
	20-24	101	26.4
	25-29	142	37.1
	30-34	113	29.5
	35 and above	2	0.5
Religion	Orthodox	267	69.7
	Protestant	104	27.1
	Muslims	12	3.1
Maternal Educational status	No education	213	55.6
	Primary education and above	170	44.4
Marital status	married	371	96.9
	Unmarried	12	3.1
current occupation	Housewife	144	37.6
	Farmer	113	29.5
	Merchant	65	16.9
	Government employee	46	12.0
	Students	10	2.6
	Daily workers	5	1.3
Ethnicity	Bench	155	40.5
	Amhara	165	43.1
	Gurage	42	10.9
	Keffa	16	4.2
	Others*	5	1.3
Residence	Rural	200	52.2
	Urban	183	47.8
Wealth Index	Low	155	40.4
	Middle	167	43.6
	Better off	61	16
Parity	One	86	22.4
	Two –four	215	56.1
	Five and above	82	21.5
Age of the child in month	<6	31	7.9
	6-11	152	39.9
	12-23	133	34.7
	24 and above	67	17.5
Past Experience of diarrheal disease in under five children in the family.	Yes	91	23.7
	No	292	76.3

Note: * Tigray= 3, Wolayita=2

**Knowledge of diarrheal disease among lactating mothers:** The participants' level of knowledge was grouped as low, moderate and high level with (72.2%), 17.7% and 10.1% proportions of participants respectively. Knowledge of lactating mothers regarding diarrhea in which 189 (49.3%) mothers considered loose and watery stool as diarrhea. Regarding causes of diarrhea, 159(41.5%) of mothers said teething, only 19(4.9%) considered evil eyes, about signs of dehydration 209 (54.5%) did not know about it. One hundred ninety-nine respondents (51.9%) responded that diarrhea causes lethargy (Table 2).

**Table 2:** Knowledge of lactating mothers in Mizan-Aman district, South-West Ethiopia, March, 2013 (n=383)

Variable	category	Frequency	Percent
Diarrhea	Watery stool	189	49.3
	Increase frequency	103	26.9
	Both	91	23.8
Cause of Diarrhea	Contaminated water	57	14.9
	Eating mud	101	26.4
	Contaminated water and eating mud	44	11.5
	Teething	159	41.5
	Evil eyes	19	4.9
	Don’t know	3	0.8
Sign of dehydration	Sunken eyes	67	17.5
	Thirsty and dry skin	107	27.9
	Don’t know	209	54.5
Consequences	Lethargy	199	51.9
	Loss of Weight	165	43.0
	Unconsciousness	9	2.3
	Death	10	2.6
Treatment Preference during diarrhea	ORS	100	26.1
	Herbs(Traditional Medicine)	221	57.7
	Others(Porridge, Soup, soft drinks	62	16.2
Prevention	Boiling water	59	15.4
	Properly covering food	35	9.1
	Washing hand, clean environment personal hygiene	277	72.3
	Don’t know	12	3.2

Similar finding was observed from in-depth interview, almost all participants knew diarrhea by its Amharic name *"Tekimat"*. Watery stool was considered as diarheae which is most commonly cited by the respondents. The teething or the growing of milk teeth *"Yewetet Teris"* and eating mud is repeatedly cited cause of diarrhea. On the consequence most of key informants stated that lethargy *"Akim matat"* was raised by most of the in-depth interview participants. This finding also supported by *"if my child experiences watery stool, the first thing I am going to suspect is his milk teeth are start to grow and I am going to observe the growth of teeth by opening his mouth, if not I suspect that he may had eaten mud. If the diarrhea is persistent, his body becomes weak and immediately I consult my husband to take him to health center".* Lactating Mother, age 34 in-depth interview participant at Shahaka kebele. Regarding prevention of diarrhea, the majority of the lactating mothers 277(72.3%) knew various preventive methods like washing hands, keeping the environment and the child clean. Most of the mothers (65.5%) knew how to prepare ORS correctly. Two hundred twenty-one (57.7%) of the lactating mothers had treatment preference of herbs which is traditional healer. Among the lactating mothers has preference of diet as treatment of diarrhea, which was constituted 62(16.2%). Almost all in-depth interview participants agree on keeping mothers and child hygiene and seeking early treatment such as saving life and preventing further complication. Economic problems and distance from health facility are among the commonly reported reasons for delay to medical care seeking. Some hesitate to go to health facility early hoping that a child would get better. Thirty years old lactating mothers in-depth interview participant from Edget kebele said that, *"When my child defecates watery diarrhea, I decide to go to health center for treatment because it might result in sever complication and may result losing life of my child"*. Some different opinions also identified during in-depth interview *"My child has beads "Doka" in his neck and that keeps him from evil eye and diarrhea. Beads in his neck prevent him from diarrhea and its consequence*. (40 Years, Lactating mother at Boja).

**Perceived susceptibility of children toward diarrheal disease:** We used eleven items to assess perceived susceptibility of children getting diarrhea on lactating mothers, maximum and minimum scores were 16 and 47 with mean of 32.5. Two hundred one (52.5%) of study participants had perceived susceptibility to diarrhea score above mean and 182(47.5%) of study participants were scored below mean. In in-depth interview; more than three fourth of the participants perceived that their child might get diarrhea. They mentioned conditions that increase susceptibility to diarrhea were presence of unsafe drinking water, teething, eating soil of their children. Poor personal hygiene of mother, child and environmental sanitation. Moreover, the following opinion may support this. *"I think my child is susceptible to diarrhea, due to lack of treated and pipe line water. I am government worker I spent eight hours in the office and when I am at office and the servant at home give care to my child. Since she has another duty in home like cooking and allow the child play on the ground, this time child has the chance of eating soil. Four month ago she was severely ill due to diarrhea".* (36 years, Lactating mother at Berges)

**Perceived seriousness of diarrheal disease:** Nine items were used to assess perceived seriousness of diarrhea as child diseases. Minimum and maximum score of this construct were 17 and 35 with mean and standard deviation of 16.94+ 4.27. About 53.4% of study participants were scored above the mean and the remained 46.6% scored below the mean. In our in-depth interview, all participants ranked diarrhea as a leading health problem. Risks attributed to diarrhea were death, illness, physical weakness, consumption of time and money. *"Diarrhea is dangerous disease, if treatment were not obtained, it can cause death, it can disturb the family"* (25 years old, lactating mother at Kometa said it to explain the seriousness of the problem).

**Diarrheal disease source of information:** Of the total, 229(59.9%) study participants were reported that as they got message concerning diarrheal disease. Large proportion 165 (72.0%) of them considered Health Extension Workers as credible source of information. About 91(23.75%) of respondents were reported that one of their child had ever got diarrheal disease.

**Multivariate analysis:** In the multivariable analysis Hosmer and Lemeshow goodness of fit test was performed and it was found that the model was good (p-value = 0.27). Finally, residence, educational status, wealth index parity, perceived susceptibility perceived severity and lactating mothers had significant association at (P-value < = 0.05). According to the Multiple logistic regression lactating mothers living in urban were 4.79 times more likely experienced diarrheal disease in one of their child ((AOR=4.79,95% CI (1.33,7.78)) than mothers living in Urban areas. Lactating mothers' with no education were 28% more likely past experience diarrheal disease of their child in their household than those who had primary education and above ((AOR=0.72 95% CI(0.55,1.29)).Regarding the wealth index of the lactating mothers, low indexed lactating mothers had 8.9 times more likely had past child diarrheal experiences than those with better wealth index ((AOR = 8.9 CI(0.99, 17.45)).Lactating mothers' with perceived susceptibility and perceived severity above the mean score were 44% and 24% less likely had past child diarrheal experience when compared with those below the mean score at ((AOR = 0.44 CI(0.33,11.33)) and (AOR = 0.24CI(1.23,7.99)) respectively. Concerning the knowledge of the lactating mothers', mothers who had low knowledge related to diarrhea disease were 2.34 times more likely encountered past child diarrheal disease experience ((AOR = 2.34 CI (1.2-4.3)) (Table 3).

**Table 3:** Independent predictors of perception in diarrheal disease of Lactating mothers in Mizan-Aman district, Bench Maji Zone, South-West Ethiopia, March, 2013 (n=383)

S/N	Variables	Past diarrhea experience in childhood in the household		Crude OR (95%CI)	Adjusted OR(95%CI)	P-Value
		**Yes**	**No**			
**1**	**Residence**					
	Rural 200	58	142	2.15 (1.35,3.78)	4.79 (1.33,7.78)	0.003*
	Urban 183	33	150	1.00	1.00	
**2**	**Maternal Educational status**					
	No education 213	79	134	1.00	1.00	0.045*
	Primary education and above 170	12	158	3.01(0.09,6.99)	0.72(0.55,1.29)	
**3**	**Wealth Index**					
	Low 155	66	89	4.33(2.33,10.25)	8.9 (0.99,17.45)	0.001*
	Middle 167	20	147	1.00	1.00	
	Better off 61	5	56	0.88(0.01,8.99)	0.25 (0.01,2.33)	
**4**	**Perceived Susceptibility**					
	<Mean score182	72	110	1.00	1.00	0.001*
	>Mean score201	19	182	1.2 (0.49,1.99)	0.44 (0.33,11.33)	
**5**	**Perceived seriousness**					
	<Mean score 178	69	109	1.00	1.00	0.033*
	>Mean score 205	22	187	187	0.24 (1.23,7.99)	
**6**	**Knowledge on Diarrheal Disease**					
	Low276	55	221	2.49 (1.60-3.89)	2.34 (1.2-4.3)	0.023*
	Moderate68	30	38	1.00	1.00	
	High 39	6	33	10.1 (5.24-19.12)	0.32 (1.6-6.2)	

## Discussion

This study was conducted with the objective of assessing diarrheal disease preventive behavior among lactating mothers' and its determinant factor using behavioral model of Health Belief Model. Based on that, we attempted to broaden our understanding on household's knowledge, perception and preventive practices. In this study, level of knowledge as low, moderate and high level with (72.2%), 17.7% and 10.1% proportions of participants respectively. Concerning causes of diarrhea, 159(41.5%) of mothers said teething, only 19(4.9%) considered evil eyes, about signs of dehydration 209 (54.5%) did not know about it. 199 (51.9%) mother said diarrhea causes lethargy. This find is lesser than the study done in Nigeria and South Mozambique [3, 11]. This discrepancy might be due to level of awareness and urbanization since most of our study participants were illiterate and live in rural area. But our finding was consistence with study result of Eritrea regarding the knowledge of the lactating mothers [4]. In this study most of the lactating mothers' (65.5%) knew how to prepare ORS correctly. Two hundred twenty-one (57.7%) of the lactating mothers had treatment preference of herbs which is traditional healer. Among the lactating mothers has preference of diet as treatment of diarrhea, which was constituted 62(16.2%).The finding of this study is consistent with the EDHS 2011 survey and study done in Assosa district Ethiopia of in the rural and lesser than urban than the urban areas this difference may be due to difference sources of information richness in the urban areas [6, 9]. In total, 229(59.9%) of study participants had heard of diarrheal disease; this result is consistent with study finding of Eritrea 60.1% of study participants ever heard of diarrheal disease [4]. About 37.9% of our study participant knew the correct cause of diarrheal disease and 41.7% attributed to child teething as cause diarrheal disease. It is higher than study result of, Nigeria which is 3.2% of participants able to mention it as cause diarrheal disease [10].

The residence of the lactating mothers' living in urban were 4.79 times more likely experienced diarrheal disease in one of their child ((AOR = 4.79,95% CI (1.33,7.78)). This finding is also consistent with the study done in Benshangul-Gumuz [12]. This is because lactating mothers' living in urban areas were probability of getting more information than rural areas. Finding from this study shows that educational status of mothers was significantly associated with the past experience of diarrheal disease at the household level. Those mother with elementary and above were less past child diarrheal experience when compared with no Education. The finding is similar with the study done in Ethiopia and Eritrea [7]. Regarding the wealth index of the lactating mothers, low indexed lactating mothers had 8.9 times more likely had past child diarrheal disease experiences than those with better wealth index. The finding of the study is similar to other studies done in Ethiopia [8] and contrary to the study done in India, were income of the lactating mothers were not associated this may be socio-economic difference between two countries [13]. This study revealed the importance of diarrheal disease related knowledge was significantly associated with the past experience of diarrheal disease in the household, the finding of this study is consistent with the study done in Ethiopia, Eritrea, India [14, 15]. This study also shown that the perceived susceptibility and perceived severity of lactating mothers above the mean score were associated with their past diarrheal disease experience. Those lactating mothers with high perceived susceptibility and severity had less likely their child experienced diarrheal disease at household level. The finding of this study is consistent with the study done in India and contrary to the study done in Ghana this difference may due to socio-cultural and socio-economic status [16, 17].

## Conclusion

This study had identified important perceptions and socio-economic that contribute to the lactating mothers' diarrheal disease prevention towards their children. Perception of diarrheal disease was significantly associated with place of residence, wealth index, educational status, Knowledge, perceived susceptibility and severity of the lactating mothers. To conclude this study, perception towards the diarrheal disease were significantly associated with place of the residence, wealth index, educational status, Knowledge, perceived susceptibility and severity among lactating mothers. Effective information educational communication (IEC) programs that targeted to increase perceived susceptibility and severity of diarrheal disease by emphasizing on the benefit of adhering nationally recommended practice to prevent diarrheal disease. Health extension workers (HEWs) educating households on sanitation through their house-to-house visits. Provision of continuous information improves the environmental conditions at household levels. Encouraging female school enrolment should be strengthened.

### What is known about this topic

- The risk factors for the diarrheal diseases are studied in most countries in Africa;
- The Knowledge, attitude and practice related to the diarrheal disease are also studied in most African countries including Ethiopia;
- The factors like maternal educational status has significant association in the prevention of the diarrheal studies. This is evidence from most studies done in Ethiopia, Eritrea and Nigeria.

### What this study adds

- There is generally significant association of mothers perceived susceptibility and diarrheal diseases preventions in this study;
- Also the perception of mothers about the seriousness of diarrheal disease (i.e. perceived severity) has significant in the prevention of diarrheal diseases. Therefore, intervention and awareness raising targeting severity of the disease is more effective;
- This study employed mixed method study, which explored the perceptions of the mothers. So that this is more important to have depth understanding toward the disease for the community based belief intervention that harms child health.

## Competing interests

The authors declare no competing interests.
